# Oleomargarine, or Suet Butter

**Published:** 1875-08

**Authors:** 


Oleomargarin, or Suet Butter.—One of the most senseless things ever done by the California press was the crying down of oleomargarin and driving away its manufacture. There was not a shadow of reason for this course, and the result was accomplished by appealing to popular prejudice, and particularly by harping on the epithet—*bull butter.* The idea was propagated that it was made from dirty tallow, whereas the freshest and cleanest tallow is required—such, for instance, as is devoured without hesitancy by the daintiest people, under the name of suet, in plum-pudding and mince-pie. The article made in San Francisco was quite as good as the average of winter butter, as we know from actual trial; and it is asserted that the process of manufacturing it has been much improved since that time. It appears that the people of the Atlantic States place a very different estimate on it from ours, and that they encourage its production for several reasons; among others, because it tends to keep down the price of butter in the season of greatest scarcity, and thus to enable the poorer classes to procure it when they could not otherwise do so. Within a year past the business has assumed important proportions in the Northern States and in Canada. At Hamilton, Canada, there is a manufactory from which 2000 pounds are shipped weekly to the United States, England and the West Indies. An invoice of eight tons was lately shipped to Jamaica from New York by Pim, Forwood & Co. It preserves it sweetness much better than natural butter, and on this account is in great demand in the tropical islands and Brazil. At Boston the factory of Merriam & Co. turns out several thousand pounds a week of an article said to be better than that made in Canada. This is mostly shipped to the West Indies. * * *

The extent of the trade in suet butter may be inferred from the fact that more than eighty applications for patents for different processes of manufacturing it have been made, which are substantially the same as the original method devised by Hippolite Mege, a scientific investigator of Paris, who was employed by the French Government for the purpose. The New York papers say the article is regularly served at the tables of some of the best hotels in that city, and has not been detected by the guests. Immense amounts of it are palmed off for genuine butter, and if it answers the same purpose and cannot be distinguished from the genuine article, the imposition appears to us to be entirely harmless.—*Pacific Medical and Surgical Journal.*

